# Management of advanced ovarian cancer in Spain: an expert Delphi consensus

**DOI:** 10.1186/s13048-021-00816-x

**Published:** 2021-05-26

**Authors:** Andres Redondo, Ana Oaknin, Maria Jesus Rubio, Maria-Pilar Barretina-Ginesta, Ana de Juan, Luis Manso, Ignacio Romero, Cristina Martin-Lorente, Andres Poveda, Antonio Gonzalez-Martin

**Affiliations:** 1 Medical Oncology Department, Hospital Universitario La Paz-IdiPAZ, Universidad Autónoma de Madrid, Paseo de la Castellana, 261, 28046 Madrid, Spain; 2 Medical Oncology Department, Vall d’Hebron Institute of Oncology (VHIO), Hospital Universitari Vall d’Hebron, Vall d’Hebron Barcelona Hospital Campus, Barcelona, Spain; 3 Medical Oncology Department, Hospital Universitario Reina Sofía, Universidad de Córdoba (UCO), Córdoba, Spain; 4 Medical Oncology Department, Girona Biomedical Research Institute (IdIBGi) and Department of Medical Sciences, Catalan Institute of Oncology (ICO), Medical School University of Girona, Girona, Spain; 5 Medical Oncology Department, Hospital Universitario Marqués de Valdecilla, Santander, Spain; 6 Medical Oncology Department, Hospital Universitario 12 de Octubre-i+12, Madrid, Spain; 7 Medical Oncology Department, Instituto Valenciano Oncologia, Valencia, Spain; 8 Medical Oncology Department, Hospital Universitario de la Santa Creu i Sant Pau, Barcelona, Spain; 9 Oncogynecologic Department, Initia Oncology, Hospital Quironsalud, Valencia, Spain; 10 Medical Oncology Department, Clínica Universidad de Navarra, Madrid, Spain

**Keywords:** Ovarian cancer, Advanced disease, Recurrent disease, Management, Consensus

## Abstract

**Background:**

To determine the state of current practice and to reach a consensus on recommendations for the management of advanced ovarian cancer using a Delphi survey with a group of Spanish gynecologists and medical oncologists specially dedicated to gynecological tumors.

**Methods:**

The questionnaire was developed by the byline authors. All questions but one were answered using a 9-item Likert-like scale with three types of answers: frequency, relevance and agreement. We performed two rounds between December 2018 and July 2019. A consensus was considered reached when at least 75% of the answers were located within three consecutive points of the Likert scale.

**Results:**

In the first round, 32 oncologists and gynecologists were invited to participate, and 31 (96.9%) completed the online questionnaire. In the second round, 27 (87.1%) completed the online questionnaire. The results for the questions on first-line management of advanced disease, treatment of patients with recurrent disease for whom platinum might be the best option, and treatment of patients with recurrent disease for whom platinum might not be the best option are presented.

**Conclusions:**

This survey shows a snapshot of current recommendations by this selected group of physicians. Although the majority of the agreements and recommendations are aligned with the recently published ESMO-ESGO consensus, there are some discrepancies that can be explained by differences in the interpretation of certain clinical trials, reimbursement or accessibility issues.

## Background

Worldwide, ovarian cancer comprised 3.4% of all new cases of cancer in women in 2018 [1]. Despite the improvement of survival in recent decades among patients with advanced disease [2], ovarian cancer remains the leading cause of death among gynecological cancers in developed countries [3].

As in other oncology settings, the treatment of ovarian cancer is continuously evolving. Thus, although new treatment options for primary and recurrent ovarian cancer have improved outcomes for patients, they have also increased the complexity of the management of this condition, and several areas of controversy exist.

Although one of the objectives of clinical practice guidelines for ovarian cancer is to improve and harmonize the management of the disease and the availability of several clinical practice guidelines [4–6], considerable variation exists among countries in both the recommendations and clinical practice regarding the management of this neoplasm [7, 8]. Additionally, adherence to the clinical practice guidelines is not always optimal [9, 10], and this might have an impact on patient outcomes, including survival, as well as the efficiency of the healthcare system [7, 11, 12].

The objective of this study was to determine the state of current practice and to reach a consensus on recommendations for the management of advanced ovarian cancer using a Delphi survey with a group of Spanish medical oncologists specially dedicated to gynecological tumors.

## Results

### Response rate

In the first round, 32 oncologists and gynecologists were invited to participate, and 31 (96.9%) completed the online questionnaire. In the second round, the 31 first-round respondents were invited to participate, and 27 (87.1%) completed the online questionnaire.

### First-line management of advanced disease

Detailed responses to all questions in this section, including the location, proportion, and strength (i.e., median) of consensus, are presented in Table 1.

**Table 1:**
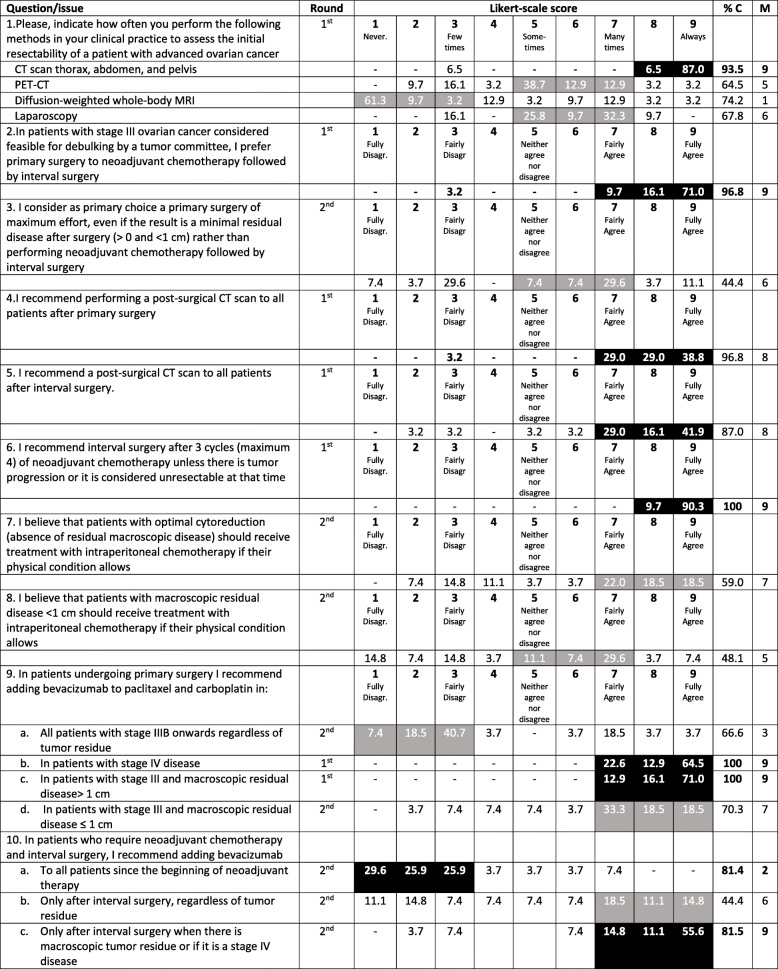
First-line management of advanced-stage ovarian cancer

*% C* proportion of consensus, *CT* Computed tomography, *M* Median, *MRI* Magnetic resonance imaging, *PET-CT* Positron emission tomography–computed tomography

Black shadowed areas represent the presence of consensus (i.e., ≥75% of the responses are located within those three consecutive points of the Likert scale)

Gray shadowed areas represent the triplet with a greater proportion of responses but without reaching consensus threshold

The initial assessment for resectability always includes computed tomography (CT) of the thorax, abdomen and pelvis according to the vast majority of the respondents. Positron emission tomography (PET)-CT and laparoscopy are sometimes used (median 5 and 6, respectively), while diffusion-weighted whole-body magnetic resonance imaging (MRI) is rarely performed (median 1). There was consensus for recommending a postsurgical CT scan for all patients both for those undergoing primary surgery and those undergoing interval surgery (median 8 in both cases).

**Fig. 1:**
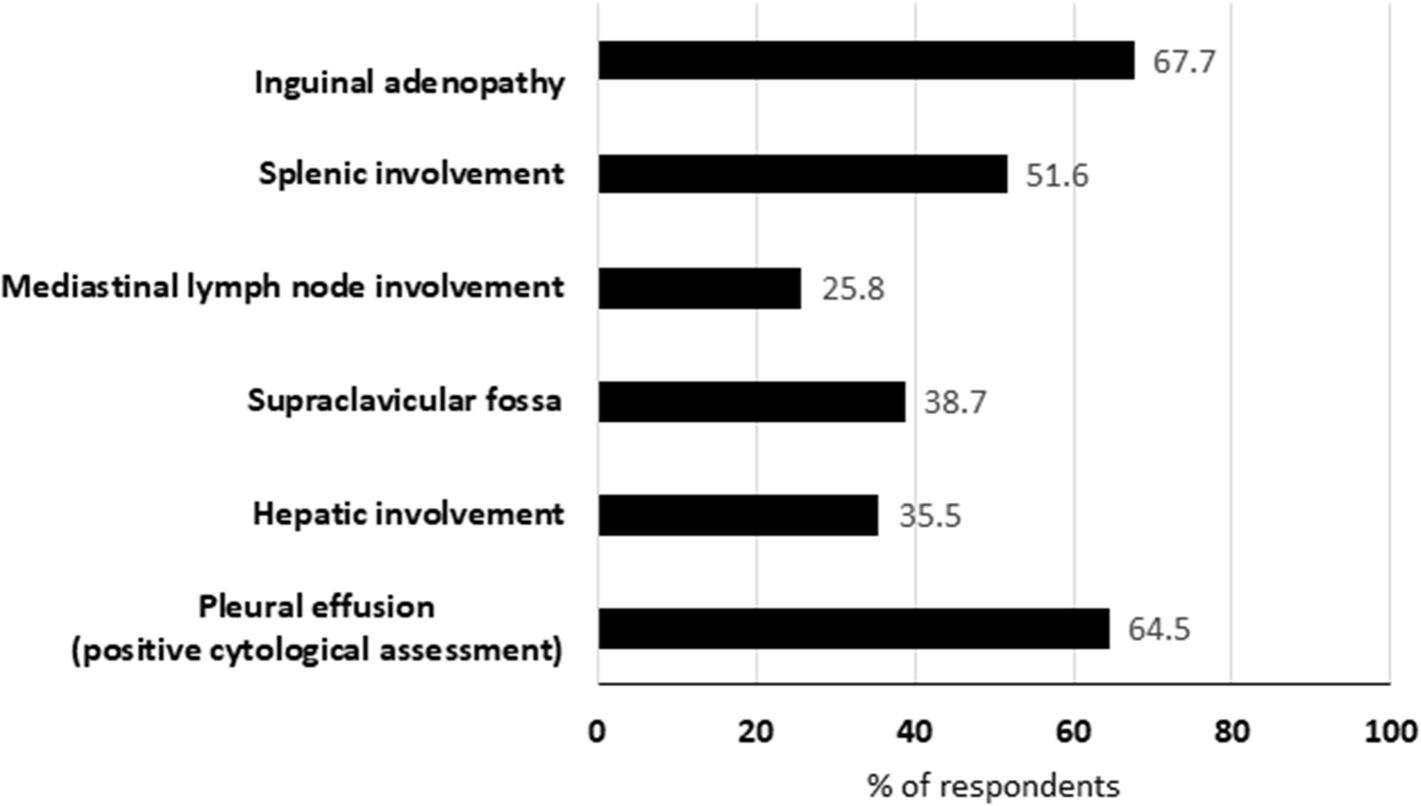
Preference of primary debulking surgery over neoadjuvant chemotherapy in different stage IV scenarios

There was consensus in using primary debulking surgery (PDS) instead of neoadjuvant chemotherapy (NACT) followed by interval debulking surgery (IDS) for patients with stage III disease considered suitable for primary debulking (median 9), but consensus was not reached for selecting this strategy as the primary strategy when minimal residual disease is expected to be left after surgery (median 6). Regarding patients with stage IV disease, if they are considered suitable for debulking surgery, two-thirds of the respondents preferred to start with PDS instead of NACT in the subgroup of patients with inguinal adenopathy or patients with positive pleural effusion (Fig. 1). A consensus with a high degree of agreement (100% responding 8–9, median 9) was reached in recommending IDS after NACT unless there is tumor progression or the tumor is considered unresectable.

There was no agreement for recommending intraperitoneal chemotherapy for either of the two proposed clinical scenarios. However, an important proportion of participants (59%) fairly to fully agreed (median 7) to recommend it for patients with optimal cytoreduction if their physical condition allows it.

Regarding the addition of bevacizumab to paclitaxel and carboplatin, the respondents agreed to recommend this approach in patients who underwent primary surgery if they have stage IV (median 9) or stage III cancer with macroscopic residual disease > 1 cm (median 9). Similarly, there was agreement in recommending adding bevacizumab after IDS for patients who require NACT when there is macroscopic residual disease or for stage IV patients (median 9).

### Treatment of patients with recurrent disease for whom platinum might be the best option

The specific responses to the 13 questions of this section and measures of consensus are presented in Table 2.

**Table 2:**
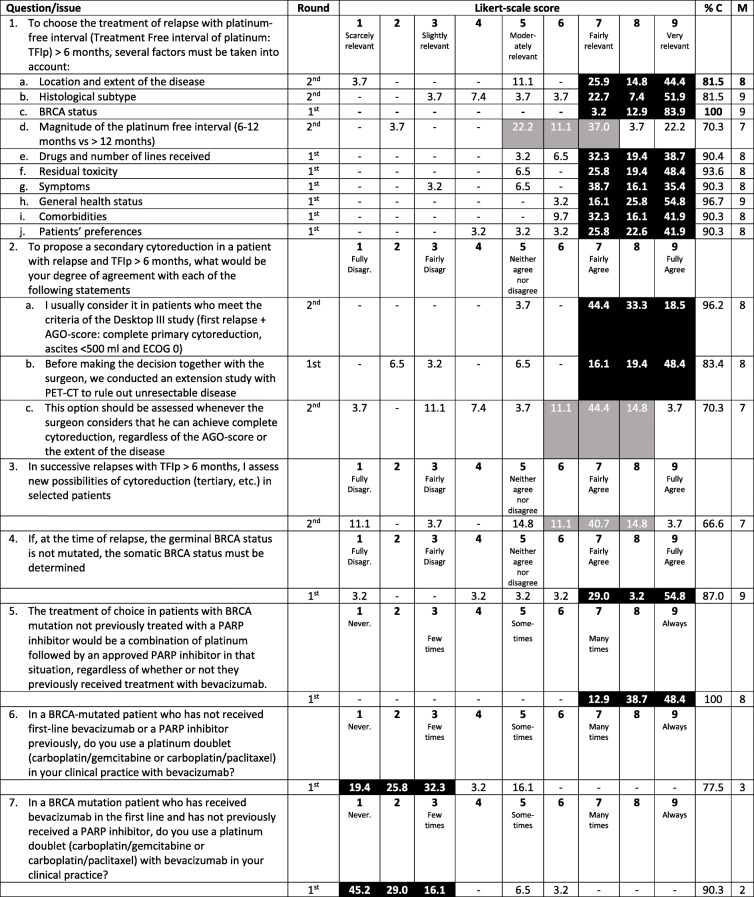

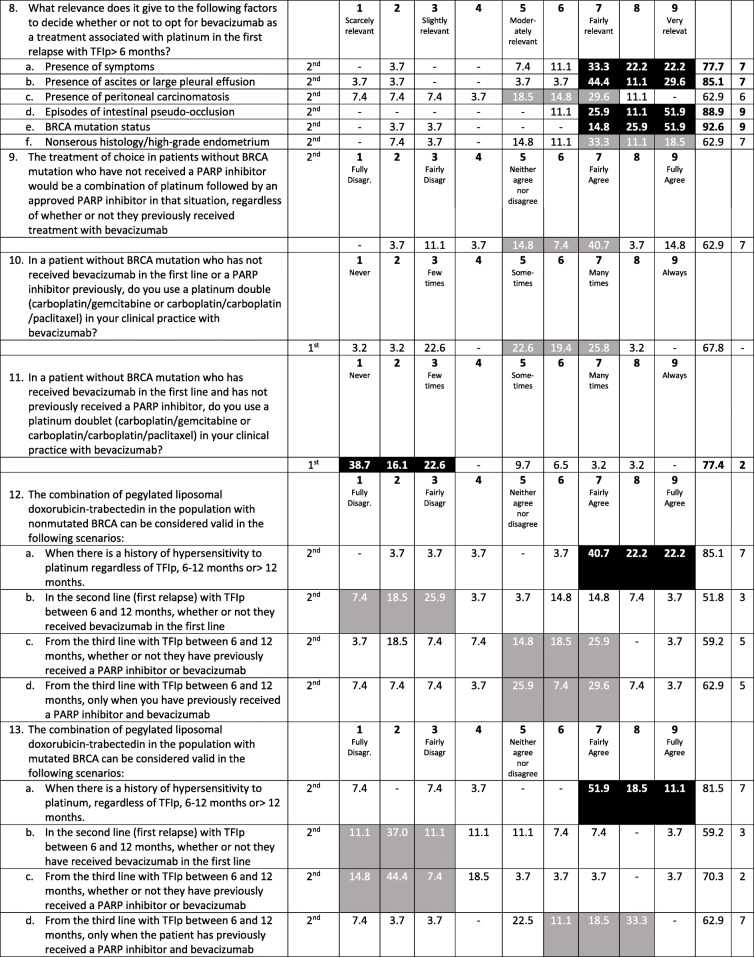
Recurrent ovarian cancer: treatment of patients eligible for platinum

*AGO* Arbeitsgemeinschaft Gynäkologische Onkologie (German Working Group on Gynecological Oncology), *% C* proportion of consensus, *ECOG* Eastern Cooperative Oncology Group, *M* Median, *PARP* Poly(ADP-ribose) polymerase, *PET-CT* Positron emission tomography–computed tomography, *TFIp* Treatment-free interval of platinum

Black shadowed areas represent the presence of consensus (i.e., ≥75% of the responses are located within those three consecutive points of the Likert scale)

Gray shadowed areas represent the triplet with greater proportion of responses but without reaching the consensus threshold

For selecting the treatment for patients with a treatment-free interval of platinum (TFIp) exceeding 6 months, there was consensus among respondents that the following 9 factors, ordered by degree of relevance, should be taken into account: *BRCA* status, histological subtype, performance status (median 9 for these 3 factors), location and extent of the disease, drugs already received and number of prior lines, residual toxicity, symptoms, comorbidities and patient preferences (median 8 for these 6 factors). The magnitude of the TFIp (6–12 vs > 12 months) was not considered a very relevant factor (median 7).

A consensus was reached (median 9) to recommend somatic *BRCA* testing at the time of relapse for patients with germline nonmutated *BRCA* if somatic testing had not been performed at diagnosis.

The participants agreed that for patients with recurrent disease and a TFIp exceeding 6 months, a secondary cytoreductive surgery should be considered if the patient meets the so-called AGO (Arbeitsgemeinschaft Gynäkologische Onkologie- German Working Group on Gynecological Oncology) score used in the DESKTOP III trial (ECOG 0, complete resection at primary surgery and the absence of large volume [> 500 mL] ascites) and after assessment of the extension using PET-CT to rule out unresectable disease (median 8 in both cases). In patients with subsequent relapse after secondary cytoreductive surgery and a TFIp > 6 months, there was no agreement in considering tertiary cytoreductive surgery for selected patients (median 7).

In the *BRCA*-mutated recurrent OC population, platinum-based chemotherapy followed by a PARP inhibitor (PARPi) was considered the treatment of choice if the patients have not previously been treated with PARPi, regardless of previous treatment with bevacizumab (median 9). Consistently, for these patients, the combination of a doublet containing carboplatin with bevacizumab is infrequently used (median 3).

In the *BRCA* nonmutated population who has not received a prior PARPi, regardless of previous treatment with bevacizumab, there was no consensus for recommending platinum-based chemotherapy followed by a PARPi or platinum-based chemotherapy with bevacizumab. The latter option is infrequently used for patients who have received first-line treatment with bevacizumab.

The respondents agreed that the following factors are relevant for deciding the addition of bevacizumab to platinum-based chemotherapy: *BRCA* mutation status (median 9), history of intestinal subocclusion (median 9), presence of ascites or large pleural effusion (median 7), and presence of symptoms (median 7).

The combination of pegylated liposomal doxorubicin with trabectedin was only recommended as a valid strategy for patients with a history of platinum hypersensitivity regardless of the TFIp (6–12 vs > 12 months) and *BRCA* status (median 7 for *BRCA* mutated and nonmutated).

### Treatment of patients with recurrent disease for whom platinum might not be the best option

The results of the 7 questions for this section are presented in Table 3.

**Table 3:**
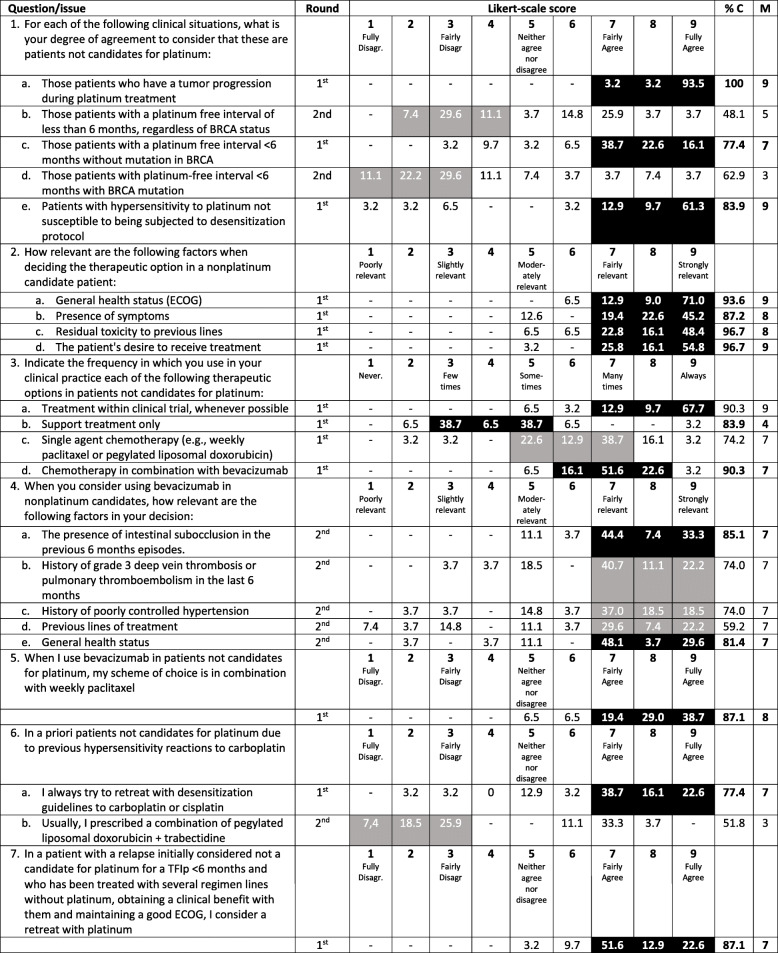
Recurrent ovarian cancer: treatment of patients non-eligible for platinum

*% C* proportion of consensus, *ECOG* Eastern Cooperative Oncology Group, *M* Median, *TFIp* Treatment-free interval of platinum

Black shadowed areas represent the presence of consensus (i.e., ≥75% of the responses are located within those three consecutive points of the Likert scale)

Gray shadowed areas represent the triplet with greater proportion of responses but without reaching the consensus threshold

There was consensus that patients could be considered ineligible for platinum in the following clinical situations: patients who have had tumor progression during platinum treatment (median 9), patients who have shown hypersensitivity to platinum and are not candidates for a desensitization protocol (median 9), and patients with a TFIp < 6 months without a *BRCA* mutation (median 7). One-third of the participants showed a high degree of agreement that a TFIp < 6 months by itself defines ineligibility for platinum regardless of the *BRCA* mutation status, while another third of the respondents disagreed with this statement (median 5).

Factors considered more relevant (median degree of relevance 8–9) for determining the therapeutic option for patients who are ineligible for platinum were performance status, the patient’s desire, residual toxicity and the presence of symptoms. The most frequent therapeutic option used for these patients is treatment within a clinical trial whenever possible (median 9), followed by single agent chemotherapy in combination with bevacizumab (median 7). When using bevacizumab, most of the respondents agreed that the preferred chemotherapy is weekly paclitaxel (median 8).

The most relevant factors to be taken into consideration for not using bevacizumab in this population are a recent (< 6 months) history of subocclusion and poor performance status (median 7 in both cases); although the cutoff for consensus was not reached, a recent history of grade 3 deep vein thrombosis or pulmonary thromboembolism, history of poorly controlled hypertension and, to a relatively lower extent, previous lines of treatment were also considered relevant factors for not selecting bevacizumab (median 7 in all cases).

If the patient is not eligible for platinum because of a history of hypersensitivity to carboplatin, the preferred approach is platinum rechallenge following a desensitization protocol (median 7). Platinum rechallenge is also the preferred option for a patient who was initially defined as ineligible for platinum due to a TFIp < 6 months and has been treated with several lines of nonplatinum regimens, showing a clinical benefit and maintaining a good performance status (median 7).

## Discussion

This expert consensus survey aimed to provide a snapshot of the current practice and expert recommendations for some key—and sometimes controversial—issues in the management of advanced ovarian cancer in Spain using a Delphi survey. Overall, we accounted for a large participation of the selected experts.

Initial extension assessment of the disease is a matter of discussion, and the latest ESMO-ESGO consensus recommends the necessity of standardization. CT, PET-CT and diffusion-weighted whole-body MRI are among the imaging techniques that can be used in the initial workup [3]. According to the experts surveyed, the CT scan is the most common test to evaluate the disease stage at diagnosis. PET-CT is used less frequently, and diffusion-weighted whole-body MRI is rarely used, probably due to the more limited access to this technique. Although laparoscopy might provide a definite histopathological diagnosis and information on disease burden [3], according to our survey, it is not implemented as a first approach in all hospitals.

The goal of cytoreductive surgery must be complete cytoreduction, that is, the absence of macroscopic residual disease, since this is the most important prognostic factor in patients with advanced disease [13]. Several randomized clinical trials have shown no significant difference in progression free survival (PFS) and overall survival (OS) between PDS and IDS [14, 15]. However, most clinical practice guidelines recommend PDS when the patient has an adequate performance status and complete cytoreduction is considered feasible [3], since in these cases, the OS of patients with PDS could be longer than with IDS as reported in a retrospective study [16]. The results of this survey are consistent with this latter recommendation, and the respondents prefer PDS when the tumor is considered suitable for debulking, independent of FIGO stage III or IV. If IDS is an option, there was a strong agreement for recommending it after 3 cycles (and no more than 4 cycles) of NACT.

The combination of carboplatin and paclitaxel has been the first-line standard chemotherapy for over 20 years; other options include the administration of intraperitoneal therapy, which has been a matter of great debate, and the addition of bevacizumab as part of the first-line treatment [17]. There was no consensus for recommending intraperitoneal chemotherapy as first-line therapy, neither for patients with optimal debulking surgery nor for those with residual disease ≤1 cm. However, more than 50% of respondents agreed that patients with optimal cytoreduction may receive intraperitoneal chemotherapy if they have adequate performance status. Although the GOG 172 study showed a benefit in terms of OS with the use of intraperitoneal chemotherapy, the recent results of the GOG 252 trial have cast doubt on the superiority of intraperitoneal administration, at least when bevacizumab is added to chemotherapy [18, 19]. The results of this survey reflect the debate around this approach among the oncology community.

In our survey, there was agreement for using bevacizumab for all patients with stage IV disease, both for those undergoing PDS and for those who require NACT and IDS. However, in patients with stage III disease, bevacizumab is only widely recommended if there is macroscopic residual disease > 1 cm. The benefit of bevacizumab in stage III patients without residual disease has yet to be demonstrated, despite the results of an exploratory subgroup analysis of the ICON7 trial showing that the addition of bevacizumab to front-line chemotherapy improves PFS regardless of the stage or presence of residual disease [20]. The results of two randomized phase II trials did not show clear advantage for adding bevacizumab to neoadjuvant chemotherapy [21, 22]. However, the administration of bevacizumab after IDS might provide a benefit regardless of whether complete cytoreduction is achieved, as these patients are at higher risk of relapse due to greater tumor burden at diagnosis precluding PDS). Nevertheless, this hypothesis has not been proven, and therefore, most of the responders recommended restricting the use of bevacizumab after PDS for those patients with macroscopic residual disease.

Of note, the evaluation of the first-line treatment of this consensus was carried out without considering the recent results shown in the maintenance setting with PARPi, as at the time of modeling the first survey no PARPi had been approved in the front line [23–26].

The use of the 6-month cutoff of TFIp for defining platinum sensitivity or resistance has been abandoned in the ESMO-ESGO consensus, and a therapeutic-oriented definition is proposed classifying patients into two groups: those for whom platinum might not be the best option (defined by early symptomatic relapse, progression on prior platinum-based chemotherapy, or platinum intolerability) and those for whom platinum might be the best option or rechallenge appears justified (defined by a response to prior platinum-based chemotherapy and the absence of contraindications for platinum) [3]. Consistent with clinical guidelines, in our study the factors considered more relevant for selecting the treatment in patients with a TFIp exceeding 6 months were *BRCA* mutation status, histological subtype and performance status; the location and extent of the disease, drugs and number of lines received, residual toxicity, presence of symptoms, comorbidities and patient preferences were also considered relevant.

Aligned with the ESMO-ESGO consensus, the respondents agreed that secondary debulking surgery should be offered to motivated patients with recurrent disease and a TFIp that exceeds 6 months if they meet the AGO score of the DESKTOP III study based on the benefit on PFS and OS recently communicated [27, 28]. Additionally, it would be advisable to perform PET-CT to have a better evaluation of the disease extension and therefore increase the likelihood of successful complete surgery, as agreed upon in this consensus. Although there are some case series that show a possible benefit with subsequent cytoreductive surgeries (tertiary or quaternary cytoreduction) [29, 30], the limited evidence available has not allowed consensus to be reached on this point.

In patients for whom platinum might be the best option, there was consensus on treating patients with *BRCA* mutations with a platinum-based combination followed by maintenance with PARPi (olaparib, niraparib or rucaparib), given the relevant benefit in terms of PFS shown in the SOLO2 [31], NOVA [32] and ARIEL3 trials [33]. Moreover, an improvement in OS was shown recently in the SOLO-2 trial [34]. There was also an agreement to analyze the status of somatic *BRCA* in all patients who have a relapse when platinum might be the best option, and germline BRCA was non mutated at diagnosis.

However, there was no consensus for recommending platinum-based therapy followed by PARPi in patients without *BRCA* mutations. The ESMO-ESGO consensus states that in these cases, patients could be offered a platinum-based rechallenge either with bevacizumab or with PARPi maintenance in cases of response [3]. Platinum-based rechallenge plus bevacizumab is usually recommended for symptomatic patients for whom a rapid response is required, based on the higher response shown in the OCEANS [35] and GOG0213 [36] trials when bevacizumab is added to platinum-based chemotherapy. Consistently in our survey, there was also consensus that the presence of large ascites or pleural effusion were relevant factors for considering the option of adding bevacizumab to a platinum-based regimen in the first relapse after a TFIp > 6 months. In the remaining patients, it would be advisable to opt for treatment with a platinum-based regimen followed by PARPi maintenance, based on the benefit in all subgroups showed by niraparib and rucaparib in NOVA [32] and ARIEL-3 [33] trials, respectively.

The role of trabectedin in the management of recurrent ovarian cancer is gradually being limited, and consistent with this, there is only consensus for recommending trabectedin in combination with pegylated liposomal doxorubicin (PLD) as a valid strategy for patients with a history of platinum hypersensitivity, regardless of the pTFI and *BRCA* mutation status. Furthermore, with the availability of PARPis and their new indications, it is likely that the use of trabectedin plus PLD will be limited to patients who have received PARPi after prior platinum-based lines but still have a TFIp > 6 months, especially for those patients with *BRCA* mutated tumors for whom a subanalysis of the trial OVC-3006 has shown a benefit in OS compared to PLD [37].

Patients with recurrent ovarian cancer for whom platinum might not be the best option have a poor prognosis and obtain very limited benefits from currently available treatments. Therefore, for these patients, a clinical trial with new therapies might be one of the best options, and this was the opinion of most of the participants in our survey. When there is no option for clinical trial, the preferred therapeutic alternative according to the respondents is single-agent chemotherapy, with weekly paclitaxel being the agent of choice, in combination with bevacizumab when indicated [38]. Factors considered relevant in our survey for avoiding bevacizumab in these patients are a history of intestinal subocclusion, poor performance status, history of deep vein thrombosis or pulmonary thromboembolism, history of uncontrolled hypertension, and, to a lesser extent, several prior lines of treatment. These factors are fairly consistent with the exclusion criteria of the AURELIA trial that led to the approval of bevacizumab in this setting [39].

The results of the survey presented in this manuscript reflect therapeutic preferences on different topics of ovarian cancer treatment, expressed by a group of Spanish experts and systematized by the Delphi method. It is not possible to draw a global conclusion about the therapeutic recommendations for advanced ovarian cancer in our country due to the obvious limitation associated with the bias inherent to the selection of a limited number of participants who are considered national experts in the field. However, this survey shows a snapshot of current recommendations by this selected group of physicians. Although the majority of the agreements and recommendations are aligned with the recently published ESMO-ESGO consensus, there are some discrepancies that can be explained by differences in the interpretation of certain clinical trials, reimbursement or accessibility issues, making this type of survey a way to approach real clinical practices in specific countries.

## Methods

### Expert panel and development of the questionnaire

The scientific committee comprised three experts (AR, AO and AGM) who selected a group of medical oncologists and gynecologists throughout Spain with high expertise in the management of ovarian cancer to participate in this survey. The scientific committee, after revising the current literature and the clinical practice guidelines available at the time of the project initiation, developed a first version of the questionnaire. That version was revised by a group of 7 experts—the review committee—who provided feedback on the questionnaire to the scientific committee, who then agreed on the final questionnaire (November 2018).

The questionnaire was divided into three areas concerning the treatment of advanced ovarian cancer: first-line treatment (10 questions), treatment of recurrent ovarian cancer in patients for whom platinum might be the best option (13 questions), and treatment of recurrent ovarian cancer in patients for whom platinum might not be the best option (7 questions). All questions but one were answered using a 9-item Likert-like scale with three types of answers: frequency (1 = never, 9 = always), relevance (1 = scarcely relevant, 9 = very relevant) and agreement (1 = fully disagree, 9 = fully agree).

The project was scientifically endorsed by GEICO (the Spanish Ovarian Cancer Research Group).

### The Delphi method

The Delphi method is a frequently used system for gathering opinions in a structured way from a group of experts [40]. The key characteristics of the method are the anonymous nature of the survey and that the participants receive feedback on their answers and may adjust their initial answers to that feedback using an iterative process [40].

We performed two rounds of administering the questionnaire via the internet on a website specifically designed for the study. The participants had to register on the website using a valid e-mail address and obtain a password in advance to fill out the questionnaire; however, the e-mail addresses were only known by the data manager in charge of the website. The website provided general instructions for completing the questionnaire and feedback on the number of questions remaining to be answered.

The first round took place between December 2018 and January 2019, and 32 experts were invited to participate. The answers from the first round were analyzed (see below) and were presented to the participants in an aggregate manner in a meeting that was held on May 24th, 2019. During the meeting, the scientific committee presented the results of the first round and promoted an open discussion on those questions, where a consensus (see definition below) was not reached. To facilitate the discussion among the attendees, they presented updated information (e.g., recommendations from clinical practice guidelines, results of clinical trials) on those questions.

The second round was carried out between June and July 2019, and using the same approach as in the first round, the participants only answered questions from the first round for which a consensus could not be reached. Questions that required a frequency answer were not included in this round since they were considered to reflect a situation/pattern instead of an opinion. To this end, each participant could look up the answer provided in the first round and modify his/her answer as appropriate.

### Statistical analysis

Descriptive statistical analysis was performed using the absolute and relative frequencies of each answer in the corresponding Likert scale. Using a cutoff point described elsewhere [41], a consensus was considered reached when at least 75% of the answers were located within three consecutive points of the Likert scale. In addition to the proportion of consensus and its location on the 9-point Likert scale, the median was also calculated to determine the strength of the consensus [10].

## Data Availability

Data is available upon request to the corresponding author.

## Abbreviations

AGO: Arbeitsgemeinschaft Gynäkologische Onkologie- German Working Group on Gynecological Oncology
CT: Computed tomography
GEICO: Spanish Ovarian Cancer Research Group
HRD: Homologous recombination deficiency
IDS: Interval debulking surgery
MRI: Magnetic resonance imaging
NACT: Neoadjuvant chemotherapy
OS: Overall survival
PARPi: PARP inhibitor
PDS: Primary debulking surgery
PET: Positron emission tomography
PFS: Progression free survival
PLD: Pegylated liposomal doxorubicin
TFIp: Treatment-free interval of platinum
